# Supplementary material for: Dynamics of chikungunya virus transmission in the first year after its introduction in Brazil: A cohort study in an urban community
Source: PLoS Negl Trop Dis. 2023 Dec 27;17(12):e0011863. doi: 10.1371/journal.pntd.0011863 (PMC10775974; doi:10.1371/journal.pntd.0011863)
Supplement: S1 Table — (DOCX) [file pntd.0011863.s001.docx]

**S1 Table.** **Demographic characteristics of the population of Salvador, the neighborhood of Pau da Lima, the study site in Pau da Lima, the sample of participants in the survey conducted in Nov/2016-Feb/2017 survey, and the cohort members who participated in all surveys between Feb-Apr/2014 and Nov/2016-Feb/2017.**

| **Characteristic** | **Salvador¹** | **Pau da Lima²** | **Study site in Pau da Lima ³** | **Participants in the Nov/2016-Feb/2017 survey³** | **Cohort members who participated in all surveys between 2014 and 2016/2017^3^** |
| --- | --- | --- | --- | --- | --- |
| **Population** | 2,675,656 | 53,284 | 2,691 | 1,776 | 652 |
| **Female sex** | 53.3% | 52.5% | 54% | 57% | 60.3% |
| **Age, in years** |  |  |  |  |  |
| <14 | 20.7% | 22.7% | 8.4% | 8.9% | 21.0% |
| 15-19 | 8.1% | 8.5% | 11.6% | 12.9% | 14.3% |
| 20-49 | 52.1% | 53.2% | 58.9% | 58.7% | 46.8% |
| 50-64 | 13.0% | 11.0% | 15.1% | 13.4% | 13.0% |
| ≥65 | 6.1% | 4.6% | 6.0% | 6.1% | 4.9% |
| **Illiteracy among those >15 years old** | 5.0% | 6.4% | NA | 3.9% | 4.9% |
| **Skin color** |  |  |  |  |  |
| Mixed | 51.7% | 51.6% | NA | 40.2% | 39.2% |
| Black | 27.8% | 35.8% | NA | 53.4% | 54.8% |
| White, Asiatic, Indigenous | 20.5% | 12.6% | NA | 6.4% | 6.0% |

NOTE: NA, not available.
¹ Data obtained from the Brazilian Institute of Geography and Statistics (IBGE), 2010.

² Data obtained from the Urban Development Company of the state of Bahia (CONDER), 2010.

³ Data obtained during study site census performed by our research team, 2016-2017.
