# Supplementary material for: Dynamics of chikungunya virus transmission in the first year after its introduction in Brazil: A cohort study in an urban community
Source: PLoS Negl Trop Dis. 2023 Dec 27;17(12):e0011863. doi: 10.1371/journal.pntd.0011863 (PMC10775974; doi:10.1371/journal.pntd.0011863)
Supplement: S2 Table — (DOCX) [file pntd.0011863.s002.docx]

**S2 Table. Sensitivity analyses on the extreme situations for missing data (excluding the sample with missing results, considering the sample with missing result as all positive or as all negative) in comparison to those derived from the participants with complete data and the random imputation method used, weighted by the likelihood of a positive result for the sample with missing data.**

| **Survey period** | **(A) Positivity of the actual samples tested** | **(B) Positivity based on the available samples to test** | **(C) Positivity based on the available samples to test plus imputation of a negative result for all the missing values** | **(D) Positivity based on the available samples to test plus imputation of a positive result for all the missing values** | **(E) Positivity based on the available samples to test plus random data imputation for missing values weighting the likelihood of a positive result by the measured positivity frequency** |
| --- | --- | --- | --- | --- | --- |
|  | **Number of positive participants/Number of tested or tested plus input participant samples**  **(% of positive results for CHIKV infection)** | | | | |
| Nov/2016-Feb/2017 | 87/652 (13.3%) | 87/652 (13.3%) | 87/652 (13.3%) | 87/652 (13.3%) | 87/652 (13.3%) |
| Mar-May/2016 | 74/80 (92.5%) | 74/645 (11.5%) | 74/652 (11.3%) | 81/652 (12.4%) | 80/652 (12.3%) |
| Aug-Nov/2015 | 51/57 (89.5%) | 51/628 (8.1%) | 51/652 (7.8%) | 68/652 (10.4%) | 70/652 (10.7%) |
| Feb-Apr/2015 | 0/47 (0.0%) | 0/624 (0.0%) | 0/652 (0.0%) | 4/652 (0.6%) | 0/652 (0.0%) |

Note: Testing was initiated with the sera of participants obtained in the last survey (Nov/2016-Feb/2017), and only those participants with a positive result had the earlier samples (Mar-May/2016) tested. Similarly, those with a positive result in the samples obtained between Mar-May/2016 had the preceding samples (Aug-Nov/2015) tested. We followed this same protocol until all the samples from a given survey were negative. We assumed that if a participant had a negative result in a sample obtained in a particular survey, the previously obtained sample would also be negative and did not test the preceding samples.

**Table description:**

Given the high positivity for the tested samples from Mar-May/2016 (92.5%) and Aug-Nov/2015 (89.5%) (A column), the extreme situations of excluding the samples not tested from the study (B column) or considering all the missing samples as negative (C column) would lead to an underestimation of the true seroprevalence in these surveys. On the other hand, the extreme situation of considering all the missing samples as positive (D column) would lead to an artificial detection of infections in 0.6% of the cohort participants in Feb-Apr/2015, when actually none of the tested samples returned positive. Thus, the random imputation based on the weighted likelihood of a positive result for the missing data produced estimates that more likely represent the truth (column E). The weights used are the positivity frequencies described in column A for Mar-May/2016, Aug-Nov/2015 and Feb-Apr/2015.
