# Supplementary material for: Dynamics of chikungunya virus transmission in the first year after its introduction in Brazil: A cohort study in an urban community
Source: PLoS Negl Trop Dis. 2023 Dec 27;17(12):e0011863. doi: 10.1371/journal.pntd.0011863 (PMC10775974; doi:10.1371/journal.pntd.0011863)
Supplement: S3 Table — (DOCX) [file pntd.0011863.s003.docx]

**S3 Table. Incidence of chikungunya virus infection according to age and sex, Salvador, Brazil, 2015-2017.**

| **Age group** | **Male** | | **Female** | | **Total** | |
| --- | --- | --- | --- | --- | --- | --- |
|  | **No. of infections/No. of participants** | **Incidence (95% CI)** | **No. of infections/No. of participants** | **Incidence (95% CI)** | **No. of infections/No. of participants** | **Incidence (95% CI)** |
| **Period: Feb-Apr/2015 to Aug-Nov/2015** | | | | | | |
| ≤14 | 12/89 | 13.5 (5.5-21.4) | 8/89 | 9.0 (3.0-15.0 | 20/178 | 11.2 (6.0-16.4) |
| 15-29 | 7/69 | 10.1 (2.9-17.4) | 7/118 | 5.9 (1.6-10.2) | 14/187 | 7.5 (3.6-11.3) |
| 30-44 | 5/45 | 11.1 (1.8-20.4) | 15/94 | 16.0 (8.5-23.4) | 20/139 | 14.4 (8.0-20.7) |
| 45-59 | 4/35 | 11.4 (0.8-22.1) | 8/64 | 12.5 (4.3-20.7 | 12/99 | 12.1 (5.7-18.6) |
| ≥60 | 2/21 | 9.5 (0.0-22.2) ^1^ | 2/28 | 7.1 (0.0-16.7) ^1^ | 4/49 | 8.2 (0.4-15.9) |
| Total | 30/259 | 11.6 (7.1-16.0) | 40/393 | 10.2 (6.8-13.6) | 70/652 | 10.7 (7.6-13.8) |
| **Period: Aug-Nov/2015 to Mar-May/2016** | | | | | | |
| ≤14 | 0/73 | 0.0 | 8/89 | 9.0 (3.0-15.0) | 0/150 | 0.0 |
| 15-29 | 2/62 | 3.2 (0.0-7.6)¹ | 7/118 | 5.9 (1.6-10.2) | 5/173 | 2.9 (0.0-5.4)¹ |
| 30-44 | 1/40 | 2.5 (0.0-7.4)¹ | 15/94 | 16.0 (8.5-23.4) | 3/119 | 2.5 (0.0-5.4)¹ |
| 45-59 | 1/35 | 2.9 (0.0-8.4)¹ | 8/64 | 12.5 (4.3-20.7) | 1/94 | 1.1 (0.0-3.1)¹ |
| ≥60 | 1/19 | 5.3 (0.0-15.4)¹ | 2/28 | 7.1 (0.0-16.7)¹ | 1/46 | 2.2 (0.0-6.4)¹ |
| Total | 5/229 | 2.2 (0.3-4.1) | 5/353 | 1.4 (0.2-2.7) | 10/582 | 1.7 (0.7-2.8) |
| **Period: Mar-May/2016 to Nov/2016-Feb/2017** | | | | | | |
| ≤14 | 1/59 | 1.7 (0.0-5.0)¹ | 0/77 | 0.0 | 1/121 | 0.8 (0.0-2.4)¹ |
| 15-29 | 2/70 | 2.9 (0.0-6.8)¹ | 3/111 | 2.7 (0.0-5.7)¹ | 3/184 | 1.6 (0.0-4.0)¹ |
| 30-44 | 0/39 | 0.0 | 2/79 | 2.5 (0.0-6.0)¹ | 1/121 | 0.8 (0.0-2.4)¹ |
| 45-59 | 0/34 | 0.0 | 0/59 | 0.0 | 1/93 | 1.1 (0.0-3.2)¹ |
| ≥60 | 0/22 | 0.0 | 0/27 | 0.0 | 1/53 | 1.9 (0.0-5.6)¹ |
| Total | 3/224 | 1.3 (0.0-2.9)¹ | 4/348 | 1.1 (0.0-2.3) | 7/572 | 1.2 (0.1-2.4) |

^1^ Whenever the lower limit of the 95% confidence interval estimated with adjustment for household clustering produced a negative value, this value was replaced by zero, as a negative value is not a valid parameter for the population.
