# Supplementary material for: Dynamics of chikungunya virus transmission in the first year after its introduction in Brazil: A cohort study in an urban community
Source: PLoS Negl Trop Dis. 2023 Dec 27;17(12):e0011863. doi: 10.1371/journal.pntd.0011863 (PMC10775974; doi:10.1371/journal.pntd.0011863)
Supplement: S4 Table — (DOCX) [file pntd.0011863.s004.docx]

**S4 Table. Prevalence of chikungunya virus infection according to age and sex, Salvador, Brazil, 2015-2017.**

| **Age group** | **Male** | | **Female** | | **Total** | |
| --- | --- | --- | --- | --- | --- | --- |
|  | **No. of infections/No. of participants** | **Prevalence (95% CI)** | **No. of infections/No. of participants** | **Prevalence (95% CI)** | **No. of infections/No. of participants** | **Prevalence (95% CI)** |
| **Period: Aug-Nov/2015** | | | | | | |
| ≤14 | 12/89 | 13.5 (5.5-21.4) | 8/89 | 9.0 (3.0-15.0 | 20/178 | 11.2 (6.0-16.4) |
| 15-29 | 7/69 | 10.1 (2.9-17.4) | 7/118 | 5.9 (1.6-10.2) | 14/187 | 7.5 (3.6-11.3) |
| 30-44 | 5/45 | 11.1 (1.8-20.4) | 15/94 | 16.0 (8.5-23.4) | 20/139 | 14.4 (8.0-20.7) |
| 45-59 | 4/35 | 11.4 (0.8-22.1) | 8/64 | 12.5 (4.3-20.7 | 12/99 | 12.1 (5.7-18.6) |
| ≥60 | 2/21 | 9.5 (0.0-22.2) ^1^ | 2/28 | 7.1 (0.0-16.7) ^1^ | 4/49 | 8.2 (0.4-15.9) |
| Total | 30/259 | 11.6 (7.1-16.0) | 40/393 | 10.2 (6.8-13.6) | 70/652 | 10.7 (7.6-13.8) |
| **Period: Mar-May/2016** | | | | | | |
| ≤14 | 10/83 | 12.0 (4.6-19.5) | 8/85 | 9.4 (3.2-15.7) | 18/168 | 10.7 (5.6-15.8) |
| 15-29 | 11/71 | 15.5 (6.9-24.0) | 9/117 | 7.7 (2.8-12.6) | 20/188 | 10.6 (5.8-15.5) |
| 30-44 | 5/44 | 11.4 (1.9-20.8) | 18/95 | 18.9 (11.0-26.9) | 23/139 | 16.5 (10.1-23.0) |
| 45-59 | 6/40 | 15.0 (3.8-26.2) | 8/67 | 11.9 (4.1-19.8) | 14/107 | 13.1 (6.7-19.4) |
| ≥60 | 3/21 | 14.3 (0.0-29.4)¹ | 2/29 | 6.9 (0.0-16.2)¹ | 5/50 | 10.0 (1.6-18.4) |
| Total | 35/259 | 13.5 (8.8-18.2) | 45/393 | 11.5 (7.9-15.0) | 80/652 | 12.3 (9.1-15.5) |
| **Period: Nov/2016-Feb/2017** | | | | | | |
| ≤14 | 9/67 | 13.4 (5.5-21.3) | 8/70 | 11.4 (4.0-18.9) | 17/137 | 12.4 (6.8-18.0) |
| 15-29 | 15/83 | 18.1 (9.1-27.0) | 9/122 | 7.4 (2.7-12.0) | 24/205 | 11.7 (6.7-16.7) |
| 30-44 | 4/43 | 9.3 (0.5-18.1) | 19/100 | 19.0 (11.3-26.7) | 23/143 | 16.1 (9.8-22.4) |
| 45-59 | 7/41 | 17.1 (5.5-28.7) | 10/68 | 14.7 (6.2-23.2) | 17/109 | 15.6 (8.4-22.8) |
| ≥60 | 3/25 | 12.0 (0.0-24.9)¹ | 3/33 | 9.1 (0.0-19.0)¹ | 6/58 | 10.3 (2.4-18.3) |
| Total | 38/259 | 14.7 (9.9-19.5) | 49/393 | 12.5 (8.8-16.1) | 87/652 | 13.3 (10.0-16.6) |

^1^ Whenever the lower limit of the 95% confidence interval estimated with adjustment for household clustering produced a negative value, this value was replaced by zero, as a negative value is not a valid parameter for the population
