# Supplementary material for: Dynamics of chikungunya virus transmission in the first year after its introduction in Brazil: A cohort study in an urban community
Source: PLoS Negl Trop Dis. 2023 Dec 27;17(12):e0011863. doi: 10.1371/journal.pntd.0011863 (PMC10775974; doi:10.1371/journal.pntd.0011863)
Supplement: Attachment — Submitted filename: 2023_09_20 Response letter. Anjos et al. PNTD.docx [file pntd.0011863.s006.docx]

 **Fundação Oswaldo Cruz**

**Instituto Gonçalo Moniz**

Rua Waldemar Falcão, 121, Candeal - CEP: 40296-710 –Bahia-Brasil

Tel: +55-71-31762289 FAX: +55-71-3176-2281

E-mail: guilherme.ribeiro@fiocruz.br

September 07, 2023

Dr. Abdallah M. Samy

Editor

PLOS Neglected Tropical Diseases

**RE:**	**Decision on your PLOS Neglected Tropical Diseases submission (PNTD-D-23-00347) - [EMID:13ff5e6cac49e45d]**

Dear Dr. Samy,

We thank you for considering our research article, "**Dynamics of chikungunya virus transmission in the first year after its introduction in Brazil: A cohort study in an urban community**". We also thank you and the reviewers for the thoughtful comments and critiques, which helped us improve the manuscript and its presentation.

As requested, we are submitting a detailed point-by-point list of responses to the comments on our article and a description of the changes we have made to the manuscript.

In addition to addressing the comments, we also included three new authors in the manuscript, as they collaborated on carrying out the plaque reduction neutralization tests and the spatial analyses.

Yours sincerely,

Guilherme de Sousa Ribeiro, MD, MS, PhD

Corresponding Author (guilherme.ribeiro@fiocruz.br)

Oswaldo Cruz Foundation, Brazilian Ministry of Health

**Title:** "Dynamics of chikungunya virus transmission in the first year after its introduction in Brazil: A cohort study in an urban community"

**Responses to Editor’s Comments**

Comment:

I agree with Reviewer 2 that it would be helpful to have a cursory description of the cohort in the Methods (e.g., recruitment approach, number of households, how/when symptom data were collected, etc). I realize that this info can largely be found in other publications, but it's helpful to have it here in sufficient detail for a reader without chasing down past publications. How large was the cohort area, and is it representative of the greater urban area?

*Response:*

*As suggested, we revised the description of the cohort in the methods section to include additional information on participants’ recruitment, number of households, and collection of data on symptoms. These details are highlighted in the 2^nd^ and 3^rd^ paragraphs of the methods section (lines 113-133).*

Comment:

Reviewer 1 requests IgM data. While I agree this data would be nice to have, this is completely optional - if you have it, great. If not, I would not expect it to be generated for this manuscript.

*Response:*

*While we agree that this data would be interesting, we appreciate your understanding that it would have a relatively minor contribution to the study relative to the costs and effort to test the samples.*

Comment:

Both reviewers were interested in more context for these results, particularly regarding reported cases in Salvador or the region of Brazil over the course of the study. If this data is available, consider adding in a figure with the time series of cases (e.g. by month), either to the main text or as a supplemental figure. However, I think Table 1 is valuable and disagree with the recommendation to modify it. Additionally, one of the reviewers requested a geographic distribution of cases - I agree this would be useful, but I would consider it optional, since I assume the MoH data would not necessarily be provided with addresses or lat/long.

*Response:*

*We thank the reviewers and the editor for these suggestions. We modified Figure 1 to include new panels with the time series of reported chikungunya cases in Salvador and the Pau da Lima community, and the geographic distribution in the study site of the participants’ households according to their immunological status of CHIKV infection. We agree that Table 1 shows valuable information and prefer not to modify it.*

Comment:

Lines 120-121: It would be helpful to include a bit more information on the use of the Euroimmun ELISA. How were positives determined, for example?

*Response:*

*We included additional information on the interpretation of the CHIKV IgG Euroimmun ELISA testing (lines 145-148) as below.*

*“The CHIKV IgG ELISA results were interpreted according to manufacturer instructions (CHIKV IgG absorbance/calibrator levels <0.8 were defined as negative, ≥0.8 to <1.1 as indeterminate, and ≥1.1 as positive). Samples with inconclusive test results were re-tested and the new results were considered final.”*

Comment:
Line 135: Having 28 out of 89 (or 87) is a large number of missing samples - this should be noted as a limitation in the Discussion. Does the random imputation potentially bias the data in some way? What seroprevalence number was used for the estimates? That should be listed here. As one of the reviewers indicated, this approach was a bit difficult to follow.

*Response:*

*Although all imputation methods are not free of the possibility of error, we believe the one we used (random imputation, weighted by the likelihood of a positive result in each survey) was the best choice to reduce bias. We now detail this procedure in the methods section to clarify how it was performed (lines 180-190). The limitation of the imputation method was cited in the original version of the manuscript. We now further emphasize that this approach may have resulted in underestimating or overestimating the incidences and prevalences measured in the surveys performed between Feb-Apr/2015 and Mar-May/2016, but not for the last survey performed in Nov/2016-Feb/2017 when all the participant samples were available for testing (lines 491-495).*

Comment:
Line 170: While OD of 0.51 is less than the mean, how does it compare to baseline and the distribution of other positive samples? It would be helpful to see the distribution of ODs in the supplemental material, or some sort of summary of the data in the Results (e.g., cutoff value was x, positive samples ranged from x to y, with a median of z, something along those lines). This goes back to the question about methods and the positive/negative cut-off value.

*Response: As suggested, we included in the manuscript the overall and survey-specific means, SD, and the minimum-maximum range of the ratios between the OD and the assay calibrator from CHIIKV IgG-positive samples (lines 250-253). To further address these unexpected ELISA results, we also performed CHIKV and MAYV plaque reduction neutralization tests (PRNT50) on samples of the two participants with potentially false-positive results obtained in Aug-Oct/2014, Feb-Apr/2014, Aug-Nov/2013, and Jan-Apr/2013. All of them were negative. We included the PRNT50 methods and results in the manuscript to support our decision to exclude these two participants from the analysis due to the false-positive CHIKV IgG signals (lines 159-171 and 253-256, respectively). We have addressed the question about how to interpret the CHIKV IgG ELISA results in a prior response.*

Comment:
Figure 1: I recommend an asterisk that says that 2 individuals that were positive earlier in the study were removed because of suspected cross-reaction. Also add in a note that results from 28 individuals were imputed.

*Response:*

*Thanks for the recommendation. We include a note with the modified Figure 1 to inform about the exclusion of two subjects with false-positive CHIKV ELISA results and the imputation of data for 28 participants.*

**Responses to Reviewers’ Comments**

**Reviewer 1:**

Comment:

Objectives clearly articulated, study design appropriate, population clearly described and appropriate. Sample size sufficient and correct statistical analysis. No ethical concerns.

*Response:*

*Thanks for the comment.*

Comment:

Results clearly presented and the figures sufficient quality for clarity.

*Response:*

*Thanks for the comment.*

Comment:

Conclusions supported by data. Limitations as noted in my major and minor comments attached. Good discussion and public health relevance addressed.

*Response:*

*Thanks for the comment.*

Comment:

Thank you for the opportunity to review this manuscript. The authors describe the dynamics of CHIKV transmission shortly after its introduction in Brazil, by performing semi-annual serosurveys in a long-term community-based cohort of 652 participants aged ≥5 years in Salvador, Brazil, between Feb-Apr/2014 and Nov/2016-Feb/2017. CHIKV infections were detected using CHIKV IgG ELISA. The cumulative seroprevalence in the last survey reached 13.3%. The authors describe a seroprevalence higher among those aged 30-44 and 45-59 years (16.1% and 15.6%, respectively), compared to younger (12.4% and 11.7% in <15 and 15-29 years, respectively) or older (10.3% in ≥60 years) age groups. The cumulative seroprevalence was similar between men (14.7%) and women (12.5%); among those aged 15-29 years, men were more often infected than women (18.1% vs. 7.4%, respectively, P = 0.01), while for those aged 30-44, a non-significant opposite trend was observed (9.3% vs. 19.0%, respectively, P = 0.12). The authors concluded that the initial spread of CHIKV in this large urban center was limited, leaving a high proportion of the population susceptible to further outbreaks.

The manuscript describes a continued important arboviral disease, CHIKV, with regional large outbreaks of disease ie Paraguay currently. The manuscript is well-written and of a topic of interest to the readers of PLOS NTD.

*Response:*

*Thanks for the comment.*

Comment:

This is a descriptive study but as a cohort study offers potential valuable insight into CHIKV disease and transmission. Important questions that could be answered relevant to the broader understanding of CHIKV is the burden of disease and symptom complex severity in children, changing mortality and morbidity, burden of chronic illness associated with acute infection, and risk of household contacts. To that end additional information and data from this study would provide insight into important questions about CHIKV:

a. Symptoms reported in table one, when obtained after infection?

*Response:*

*The self-reported symptoms were obtained during the survey performed between Nov/2016-Feb/2017. To clarify, we included this information in the second paragraph of the methods section (lines 121-125) and in a footnote for Table 1.*

b. Symptoms broken down by age in particular the pediatric population.

*Response:*

*For different reasons, we prefer not to show the reported participants’ signs and symptoms stratified by age in the manuscript. First, this characterization was not part of the main study aim, which was to describe the initial dynamics of CHIKV transmission in Salvador, Brazil. Second, we only had 87 infections detected during the cohort follow-up, of which only 17 were <15 years old. Thus, the stratification by age would result in small groups. Third, we did not detect signs and symptoms prospectively during cohort follow-up. Instead, we collected data on self-reported medical history in the last survey performed in Nov/2016-Feb/2017. As most infections occurred ~1.5 years before (between Feb-Apr/2015 and Aug-Nov/2015), recall bias is possible, as we discussed in the manuscript. Hence, pursuing the suggested analysis could lead to an overstating of the clinical findings.*

c. Deaths reported from CHIKV or hospitalizations.

*Response:*

*Unfortunately, we did not collect information on hospitalization due to chikungunya. As the cohort study design was based on participants who completed all the follow-ups, we were not able to study death occurrence because death would be a reason for loss to follow-up.*

d. An estimate of the proportion of subclinical infections in total and by age.

*Response*

*We have presented the overall proportion of CHIKV-infected participants who reported fever (37.2%), arthralgia (19.5%), or both (13.9%) in Table 1, which may suggest that most of the infections were subclinical. However, as discussed in the manuscript and the response above, recall bias associated with the study design may have underestimated these frequencies. Thus, we prefer to be cautious in interpreting it for the totality of infected cases and to refrain from pursuing additional stratified analysis by age.*

e. An estimate of household clustering of cases and risk of infection.

*Response*

*We thank the reviewer for this comment, as clustering of CHIKV infections in certain areas and within households is an important point in understanding the transmission dynamics of CHIKV. To address this comment, we* *employed the spatial scanning method using Satscan software to investigate whether community clusters occurred within the study site. We also verified whether there was an aggregation of cases within households with more than two participants by comparing the frequency of CHIKV infection among participants who resided and did not reside with someone infected with CHIKV. Interestingly, the spatial scanning identified three statistically significant clusters of CHIKV infections within the study site. In addition, the frequency of CHIKV infection among participants who lived with someone who was CHIKV IgG-positive was 45.1% (41 of 91), significantly higher than the 5.9% (21 of 358) frequency of CHIKV infection among those living with participants without CHIKV IgG antibodies (PR: 7.68; 95% CI: 3.99-14.78). These findings support prior studies suggesting that clustering of cases of CHIKV infection can be highly focal, both within certain areas and within households. We included the methods and findings relative to this investigation in the manuscript (lines 219-233 and 285-303) and also discussed them (lines 376-385).*

Comment:

Of value to mention the circulating strain of CHIKV during that time period in Salvador, I’m assuming its ECSA, or even better an isolate from the cohort study and strain.

*Response*

*Unfortunately, we did not have a CHIKV isolate from the cohort study. However, the ECSA genotype has been the only CHIKV lineage detected in the state of Bahia since the introduction of CHIKV to the country, which supports that this genotype was responsible for the infections among the cohort members. This information was already cited in the discussion section (lines 462-463).*

Comment:

Minor Comments:

1. CHIKV is a reportable disease in Brazil, it would be of value to provide context into what is being reported outside of the study area. A map of the study area within a larger map of Salvador will assist the readers in the context of the study geographic area as well as reported CHIKV for Salvador to the MoH as an epidemic curve over time during the serosurveys would be useful.

*Response*:

*As suggested, we included a map of the study site within a larger map of Salvador and also showed the location of the households of the cohort participants in the study site according to the participant’s CHIKV infection status during follow-up (Fig 1C). We agree that this addition provided geographic context to the readers and also allowed the investigation of the spatial distribution of the CHIKV infections and cases clustering. The methods and findings relative to this new analysis were included in their respective sections (lines 219-233 and 285-303) and the discussion section (lines 376-385). In addition, we included a time series of reported cases of chikungunya in Salvador and Pau da Lima (Fig 1A) and described it in lines 212-218 (methods) 281-284 (results).*

2. Geographic distribution of cases over time on the map would be useful to the spatial spread.

*Response*:

*Done, as explained in the above response (Fig 1C).*

3. Figure 2, would be useful to have an additional sub-figure showing total incidence and prevalence of CHIKV infection, according to age and sex by year.

*Response*:

*This is shown in Fig 2F, which displays the seroprevalence by age and sex in the last survey (thus, including CHIKV infections detected in Aug-Nov/2015, Mar-May/2016 and Nov/2016-Feb/2017). The seroprevalence of CHIKV infection in this last survey equals the cumulative incidence of CHIKV infection from baseline to Nov/2016-Feb/2017.*

4. The data stands by itself, but the authors could consider adding IgM to the testing (understanding the budget limitations of testing etc) as the duration of IgM following acute infection is 3 to 4 months and would provide more fidelity on the time of infection and potentially capture cases before IgG became positive ie the last serological capture point.

*Response*:

*We have addressed this comment before in a response to the editor. While we agree that this data would be interesting, we appreciate your understanding that it would have a relatively minor contribution to the study relative to the costs and effort to test the samples.*

**Reviewer 2**

Comment:

The first question is related to the so-called community-based cohort of 652 participants. Can the authors elaborate further? How many households and what was the median size of a household? Were all the members of the household recruited and followed up? What is the approx. size of the geographical area of recruitment? How were the participants approached and what was the strategy for their recruitment? Is the sample population representative for Salvador - given a very low figure for CHIKV seroprevalence?

*Response*:

*We appreciate the suggestion to add these methodological details to the manuscript. We included this information in lines 109-133 and addressed this comment before in a response to the editor.*

Comment:

I do not see a point for such a detailed Table 1. Rather, I would like to see summary statistics of the sample beside Salvador’s population statistics. Also, there should be surveillance figures, more time-granulated, for example, weekly or monthly figures in the population. How do the figures in the cohort

*Response*:

*We have addressed this comment before in a response to the editor. We believe that Table 1 shows valuable information and prefer not to modify it. However, as suggested, we included a supplementary table (S1 Table) to describe the characteristics of the populations of Salvador, Pau da Lima, and the study participants, aiming to show that the members of our cohort resemble the individuals living in the study site, which was the target population for our statistical inference.*

*Each survey lasted 3-4 months and was performed twice a year. The incidence and prevalence figures are shown for each of the surveys. We prefer not to modify this temporal scale for presenting the prevalence and incidence, as this is the typical approach used in such cohort studies. However, we now included for comparison the monthly distribution of the chikungunya cases reported in Salvador and the community of Pau da Lima between 2014 and 2017 (Fig 1A).*

**S1 Table.** Demographic characteristics of the population of Salvador, the neighborhood of Pau da Lima, the study site in Pau da Lima, the sample of participants in the survey conducted in Nov/2016-Feb/2017 survey, and the cohort members who participated in all surveys between Feb-Apr/2014 and Nov/2016-Feb/2017.

| **Characteristic** | **Salvador¹** | **Pau da Lima²** | **Study site in Pau da Lima ³** | **Participants in the Nov/2016-Feb/2017 survey³** | **Cohort members who participated in all surveys between 2014 and 2016/2017^3^** |
| --- | --- | --- | --- | --- | --- |
| **Population** | 2,675,656 | 53,284 | 2,691 | 1,776 | 652 |
| **Female sex** | 53.3% | 52.5% | 54% | 57% | 60.3% |
| **Age, in years** |  |  |  |  |  |
| <14 | 20.7% | 22.7% | 8.4% | 8.9% | 21.0% |
| 15-19 | 8.1% | 8.5% | 11.6% | 12.9% | 14.3% |
| 20-49 | 52.1% | 53.2% | 58.9% | 58.7% | 46.8% |
| 50-64 | 13.0% | 11.0% | 15.1% | 13.4% | 13.0% |
| ≥65 | 6.1% | 4.6% | 6.0% | 6.1% | 4.9% |
| **Illiteracy among those >15 years old** | 5.0% | 6.4% | NA | 3.9% | 4.9% |
| **Skin color** |  |  |  |  |  |
| Mixed | 51.7% | 51.6% | NA | 40.2% | 39.2% |
| Black | 27.8% | 35.8% | NA | 53.4% | 54.8% |
| White, Asiatic, Indigenous | 20.5% | 12.6% | NA | 6.4% | 6.0% |

NOTE: NA, not available.
¹ Data obtained from the Brazilian Institute of Geography and Statistics (IBGE), 2010.

² Data obtained from the Urban Development Company of the state of Bahia (CONDER), 2010.

³ Data obtained during study site census performed by our research team, 2016-2017.

Comment:

I am not entirely sure if I understand the method below. Why not taking extreme situations for missing data and see how sensitive the estimates might be compared to those derived on complete data.

“To reduce bias in the seroincidence estimates, rather than removing these participants from the analysis, we randomly input a CHIKV IgG status for the participants with missing data in a given survey, weighting the likelihood of a positive result by the measured seroprevalence in each of the surveys.”

*Response*:

*We thank you the reviewer for suggesting the conduction of sensitive analyses on the extreme situations for missing data (consider them as either all positive or all negative) in comparison to those derived from the participants with complete data. We did this analysis and found the following. Given the high positivity for the tested samples from Mar-May/2016 (92.5%) and Aug-Nov/2015 (89.5%) (A column), the extreme situations of excluding the samples not tested from the study (B column) or considering all the missing samples as negative (C column) would lead to an underestimation of the true seroprevalence in these surveys. On the other hand, the extreme situation of considering all the missing samples as positive (D column) would lead to an artificial detection of infections in 0.6% of the cohort participants in Feb-Apr/2015, when actually none of the tested samples returned positive. Thus, the random imputation based on the weighted likelihood of a positive result for the missing data produced estimates that more likely represent the truth (column E). The weights used are the positivity frequencies described in column A for Mar-May/2016, Aug-Nov/2015 and Feb-Apr/2015. We included the description of the methods for this analysis in the methods section (lines 190-194) and the findings as a supplementary result (S2 table).*

**S2 Table.** Sensitivity analyses on the extreme situations for missing data (excluding the samples with missing results, considering the samples with missing results as all positive or as all negative) in comparison to those derived from the participants with complete data and the random imputation method used, weighted by the likelihood of a positive result for the sample with missing data.

| **Survey period** | **(A) Positivity of the actual samples tested** | **(B) Positivity based on the available samples to test** | **(C) Positivity based on the available samples to test plus imputation of a negative result for all the missing values** | **(D) Positivity based on the available samples to test plus imputation of a positive result for all the missing values** | **(E) Positivity based on the available samples to test plus random data imputation for missing values weighting the likelihood of a positive result by the measured positivity frequency** |
| --- | --- | --- | --- | --- | --- |
|  | **Number of positive participants/Number of tested or tested plus input participant samples**  **(% of positive results for CHIKV infection)** | | | | |
| Nov/2016-Feb/2017 | 87/652 (13.3%) | 87/652 (13.3%) | 87/652 (13.3%) | 87/652 (13.3%) | 87/652 (13.3%) |
| Mar-May/2016 | 74/80 (92.5%) | 74/645 (11.5%) | 74/652 (11.3%) | 81/652 (12.4%) | 80/652 (12.3%) |
| Aug-Nov/2015 | 51/57 (89.5%) | 51/628 (8.1%) | 51/652 (7.8%) | 68/652 (10.4%) | 70/652 (10.7%) |
| Feb-Apr/2015 | 0/47 (0.0%) | 0/624 (0.0%) | 0/652 (0.0%) | 4/652 (0.6%) | 0/652 (0.0%) |

Note: Testing was initiated with the sera of participants obtained in the last survey (Nov/2016-Feb/2017), and only those participants with a positive result had the earlier samples (Mar-May/2016) tested. Similarly, those with a positive result in the samples obtained between Mar-May/2016 had the preceding samples (Aug-Nov/2015) tested. We followed this same protocol until all the samples from a given survey were negative. We assumed that if a participant had a negative result in a sample obtained in a particular survey, the previously obtained sample would also be negative and did not test the preceding samples.

Comment:

I am not sure how the authors did what they describe below – empirical methods for the cumulative prevalence and sero-incidence, yet adjusting for household clustering.
“The cumulative prevalence of CHIKV IgG antibodies was estimated for each survey by dividing the number of positive participants by the number of participants in the cohort, times 100. The CHIKV seroincidence was estimated for each survey by dividing the number of positive participants in a determined survey by the number of negative participants in the previous survey, times 100. Confidence intervals of 95% (95% CI) for the overall seroprevalences and seroincidences were estimated with adjustment for household clustering.”

*Response*:

*We apologize for not explaining it adequately before. We used Poisson regression with robust variance and clustering adjustment to estimate the seroprevalences and seroincidences with adjustment for household clustering of participants. We included this description in the methods (line 208)*

Comment:

The presentation of the data is just empirical, the panels in the figure 2 are just arithmetic quantities. The interpretation as in “men were more often infected than women…” is not appropriate, perhaps more likely. I am not entirely sure about the presentation and importance of these data. The researchers hold individual longitudinal data – yet they investigate it as aggregated data.

*Response:*

*We modified the text accordingly. Now it reads: “(…) among those aged 15-29 years, the prevalence was higher in men than women (18.1% vs. 7.4%, respectively, PR: 2.45, 95% CI: 1.21-4.95, P = 0.01) (lines 338-339).*

Comment:

I do not feel that the presented analyses match the analysis presented plan. Quite contrary, the results presentation is empirical and I do not quite understand how the authors applied their Poisson regression and how the manuscript benefited from it.

*Response*:

*Poisson regression with robust variance and adjustment for household clustering was used as a strategy to obtain more accurate 95% confidence intervals (CI) of the estimates, as the sample was not completely independent due to household clustering. 95% CIs were calculated for the seroprevalences and seroincidences and are shown in S3 and S4 Tables as well as for the prevalence ratios described in lines 338-341.*

Comment:

I do not understand what is the population the researchers aim at making inference about using their sample data - namely what the sample data represent.

*Response*:

*Our study sample was recruited in three valleys of the Pau da Lima community in Salvador in Nov/2016-Feb/2017. During the enrollment, 1,776 (67.2%) of the 1,214 inhabitants living in the study site agreed to participate in the survey and consented to have their prior serum samples tested for the presence of CHIKV IgG antibodies. However, only 654 (36.8%) of them had participated in the preceding five consecutive semi-annual cohort follow-up surveys and were analyzed as the final study sample. Additional information on the recruitment process was included in the methods section (lines 127-133). While we agree that this group does not represent a random sample of the study site inhabitants, we employed statistical inference because we considered the sample relatively representative of the study site population (which is the targeted population for the statistical inference; see prior comment and S1 Table).*

*Although the low-income population in the study site does not represent the overall population of Salvador, it is likely that it resembles the population of other low-income areas of the city and therefore we believe that our findings may be generalized to other regions of Salvador. Nevertheless, we agree that performing statistical inference using a sample that is not truly random may be considered controversial. If the reviewer and the editor feel that this is the case, we would be OK with removing the 95% CIs that we estimated for the prevalences and incidences.*
