# Supplementary material for: Dynamics of chikungunya virus transmission in the first year after its introduction in Brazil: A cohort study in an urban community
Source: PLoS Negl Trop Dis. 2023 Dec 27;17(12):e0011863. doi: 10.1371/journal.pntd.0011863 (PMC10775974; doi:10.1371/journal.pntd.0011863)
Supplement: Attachment — Submitted filename: 2023_11_15 Response letter. Anjos et al. PNTD.docx [file pntd.0011863.s007.docx]

 **Fundação Oswaldo Cruz**

**Instituto Gonçalo Moniz**

Rua Waldemar Falcão, 121, Candeal - CEP: 40296-710 –Bahia-Brasil

Tel: +55-71-31762289 FAX: +55-71-3176-2281

E-mail: guilherme.ribeiro@fiocruz.br

November 15, 2023

Brett M. Forshey

Academic Editor

Dr. Abdallah M. Samy

Section Editor

PLOS Neglected Tropical Diseases

**RE:**	**Decision on your PLOS Neglected Tropical Diseases submission (PNTD-D-23-00347R1) - [EMID:** **952976384b3d1b3a]**

Dear Dr. Forshey and Dr. Samy,

We thank you for considering our research article, "**Dynamics of chikungunya virus transmission in the first year after its introduction in Brazil: A cohort study in an urban community**". We also thank you and the reviewers for the thoughtful comments and critiques, which helped us improve the manuscript and its presentation.

As requested, we are submitting a detailed point-by-point list of responses to the comments on our article. **No additional changes were made to the manuscript**; therefore, the submitted versions of the manuscript and of the revised article with highlighted changes are the same as those previously submitted.

Yours sincerely,

Guilherme de Sousa Ribeiro, MD, MS, PhD

Corresponding Author (guilherme.ribeiro@fiocruz.br)

Oswaldo Cruz Foundation, Brazilian Ministry of Health

**Title:** "Dynamics of chikungunya virus transmission in the first year after its introduction in Brazil: A cohort study in an urban community"

**RESPONSES TO EDITOR’S COMMENTS**

Comment:

The authors have addressed the major concerns of the reviewers. While some reasonable concerns remain from one of the reviewers, I consider these to be largely related to data interpretation, and I believe the authors should have some leeway to interpret their findings.

*Response:*

*We thank the editor for this understanding.*

**RESPONSES TO REVIEWERS’ COMMENTS**

**Methods**

Reviewer #1 comment:

Authors have addressed the reviewers' comments adequately on the methodology of this manuscript.

*Response:*

*Thanks.*

Reviewer #2 comment:

The objectives of the study are reasonably narrated. The longitudinal aspect of the study aims at answering researchers' questions. The description of the population improved although it is not entirely clear whether the sample represents the population. But efforts have been made in this sense. This is an observational study aiming at describing the epidemiology of an infective agent hence an a priori sample size is not necessary.

The statistical methodology description has improved. I have no concerns about ethical or regulatory requirements.

I am not entirely sure about the title of the communication, namely transmission. I find it little relevant as the authors did not derive key dynamics elements such a force of infection which would make possible to calculate some periodic R0. Their entire analysis in merely descriptive. Also, the incidence estimates are not robust, perhaps prevalence would suffice to describe the data. It is reasonable to assume that CHIKV induces long life immunity hence the prevalence of past exposure may be a better epidemiological measure to refer to and describe the disease.

*Response:*

*Although estimating the force of infection was not one of the objectives of our study, we believe that our descriptive cohort study, performed in an urban community of a large Brazilian city, contributes to the understanding of CHIKV transmission dynamics. The study determined when transmission of the virus began, detected that it peaked six months after the initiation of transmission, in mid-2015, and revealed the establishment of endemic CHIKV circulation between 2016-2017. It suggested that there may be differences in infection risk by sex and age. Furthermore, it identified that CHIKV transmission is spatially heterogeneous, forming areas of infection clusters. Particularly relevant to public health, we also found an increased risk of infection for those who live in the same house where another CHIKV infection occurred.*

*Both incidence and prevalence are useful epidemiological indicators in population-based studies. Incidence is of special interest to describe the intensity of transmission of microorganisms that cause acute illness, such as CHIKV and other arboviruses, while seroprevalence can display the frequency of prior infection and the level of immunity and susceptibility in the population. Therefore, we preferred to maintain both of them in the manuscript.*

**Results**

Reviewer #1 comment:

Authors have addressed the reviewers' comments adequately on the analysis and resultsof this manuscript.

*Response:*

*Thanks.*

Reviewer #2 comment:

The analysis generally matched the plan. The graphical representation of the data are also more informative in this version and efforts are appreciated in this sense. I am not entirely sure about the incidence estimates. Due to low numbers, Table S3 is full of altered 95%CIs and I am not sure of their relevance. Best to present just the numbers and no estimation at all.

*Response:*

*We understand the reviewer’s point of view but prefer to keep the 95% CIs for the incidences, shown in Table S3. Censoring the 95% CI on zero is a common practice when a negative value for the lower limit of the interval is obtained but it is not a valid parameter for the population. In addition, even when the lower limit is censored, the upper limit of the 95% CIs provides information on the precision of the estimations, which may be useful information for the readers. Finally, as Table S4 shows the 95% CIs for the calculated prevalences, it is consistent that Table S3 also displays similar information for the calculated incidences.*

Reviewer #2 comment:

line 371: "...endemic transmission remained low, with ~3% of those still susceptible becoming infected". I am very sorry but this figure is not a measure of transmission. No quantitative epidemiological measures of transmission have been presented here.

*Response:*

*We respectfully disagree with the reviewer. Incidence measurements describe the frequency of new cases of a disease (or a health-related condition) in relation to a given population over time, indicating the disease risk. When the new health-related condition being studied is an infection, the incidence measurement reflects the intensity of transmission of the causative infection agent in the studied population. In this scenario, a high incidence indicates that transmission is high, while a low incidence indicates that transmission is low.*

Reviewer #2 comment:

line 519: "The low incidence and minimal herd immunity levels less than three years after the CHIKV introduction place 85% of the population at risk for new outbreaks" Do the authors have any evidence of that/ In which population? Herd immunity is strictly related to R0 and disease dynamics -it looks to me that the authors speculate on issues without evidence.

*Response:*

*Although the level of R0 is indeed a factor that influences the occurrence of herd immunity, we disagree that “Herd immunity is strictly related to R0 and disease dynamics”. Herd immunity occurs when a significant portion of the population becomes immune to an infectious agent (by vaccination or natural infection), limiting the further spread of the agent and providing indirect protection for those not yet immune. Thus, even if nothing is known about the R0 of an infectious agent, it is possible to determine the likelihood that herd immunity was reached based on the frequency of immune and susceptible individuals in a population. Although a universal cut-off level for herd immunity that could be applied to all infectious agents does not exist, it is very reasonable to assume that herd immunity was not achieved when 85% of the population does not have antibodies and is thus, susceptible to infection. Of notice, as mentioned before, herd immunity can result from mass vaccination in areas where the infectious agent was never introduced and therefore the local R0 was always zero.*

**Conclusions**

Reviewer #1 comment:

Authors have addressed the reviewers' comments adequately and the conclusion supported by the data presented.

*Response:*

*Thanks.*

Reviewer #2 comment:

I rest my case regarding the title - below is the conclusion of the authors. The title should reflect what's been done, not what needs to be done.

"Additional investigations are needed to elucidate the factors driving CHIKV spread dynamics, including understanding differences with respect to dengue and Zika viruses, in order to guide prevention and control strategies for coping with future outbreaks."

*Response:*

*As we had answered previously, we believe that our study provides relevant information related to the transmission dynamics of CHIKV in the first year after its introduction in*

*Brazil. Therefore, we preferred to keep the current title.*

**Summary and General Comments**

Reviewer #1 comment:

A much improved manuscript and of interest to the readers of PLOS NTD.

*Response:*

*Thanks.*

Reviewer #2 comment:

The paper is of interest but it still needs revision. I am happy to read it again - but there is too much from too little data and it seems that the incidence cannot be robustly estimated. There are also lots of speculative comments without references or supported by the data in the Discussion.

*Response:*

*Our study is novel in being one of the few population-based cohort studies carried out to investigate the transmission of CHIKV in the first year after its introduction into a naïve population. The incidence measurements were properly estimated. The low levels observed do not reduce its validity; conversely, they truly indicate the actual situation of a low level of CHIKV infection occurrence. We don’t see a problem in using the discussion section to propose new hypotheses and to speculate about possible explanations for the findings. Actually, that is part of what we expect to see in the discussion section of any manuscript. Our manuscript has 60 references and most of them were used in the discussion section, in order to discuss the findings and support the interpretation of results. We reviewed this section again and are confident that it was structured on the basis of the obtained results, which were discussed with the support of appropriate references.*
